# Poly[[μ-*N*,*N*′-bis­(2-hy­droxy­eth­yl)-*N*,*N*,*N*′,*N*′-tetra­methyl­propane-1,3-diaminium-κ^2^ *O*:*O*′]tetra-μ-bromido-dibromidodimanganese(II)]

**DOI:** 10.1107/S1600536812044765

**Published:** 2012-11-03

**Authors:** Heikki Rinta, Anssi Peuronen, Manu Lahtinen

**Affiliations:** a University of Jyväskylä, Department of Chemistry, PO Box 35, FI-40014 JY, Finland

## Abstract

The asymmetric unit of the title three-dimensional coordination polymer, [Mn_2_Br_6_(C_11_H_28_N_2_O_2_)]_*n*_, consists of one Mn^II^ cation, half of a dicationic *N*,*N*′-bis­(2-hy­droxy­eth­yl)-*N*,*N*,*N*′,*N*′-tetra­methyl­propane-1,3-diaminium ligand (*L*) (the other half being generated by a twofold rotation axis), and three bromide ions. The Mn^II^ cation is coordinated by a single *L* ligand *via* the hy­droxy O atom and by five bromide ions, resulting in a distorted octa­hedral MnBr_5_O coordination geometry. Four of the bromide ions are bridging to two adjacent Mn^II^ atoms, thereby forming polymeric chains along the *a* and *b* axes. The *L* units act as links between neighbouring Mn—(μ-Br)_2_—Mn chains, also forming a polymeric continuum along the *c* axis, which completes the formation of a three-dimensional network. Classical O—H⋯Br hydrogen bonds are present. The distance between adjacent Mn^II^ atoms is 4.022 (1) Å.

## Related literature

For related structures of *M* ^II^ transition metal halide one-dimensional coordination polymers, see: Han *et al.* (2012 ▶); Englert & Schiffers (2006 ▶). For two-dimensional networks, see: Hu & Englert (2006 ▶); Turgunov *et al.* (2011 ▶). For properties of metal halides, see: Hitchcock *et al.* (2003 ▶); Wang *et al.* (2011 ▶). For ligand conformations, see: Kärnä *et al.* (2010 ▶).
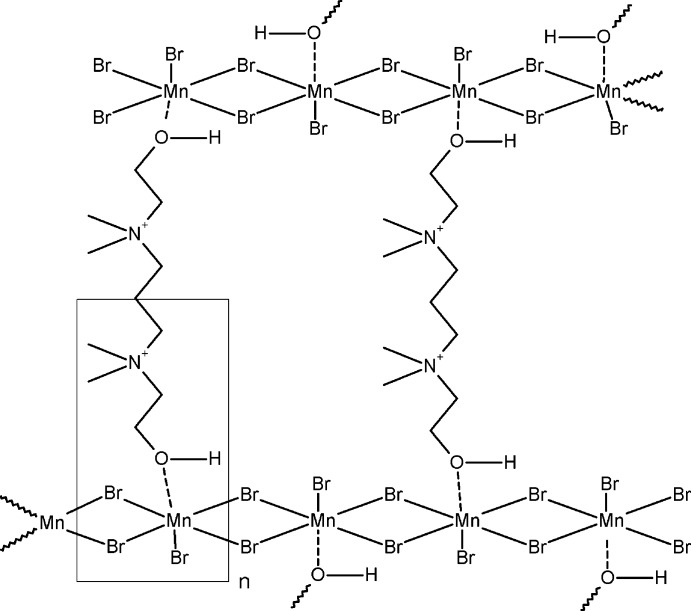


## Experimental

### 

#### Crystal data

[Mn_2_Br_6_(C_11_H_28_N_2_O_2_)]
*M* *_r_* = 809.69
Tetragonal, 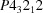

*a* = 8.0163 (4) Å
*c* = 35.3103 (18) Å
*V* = 2269.1 (2) Å^3^
*Z* = 4
Mo *K*α radiation
μ = 11.69 mm^−1^
*T* = 123 K
0.25 × 0.25 × 0.20 mm

#### Data collection

Bruker–NoniusKappa APEXII diffractometer
Absorption correction: multi-scan (*SADABS*; Sheldrick, 2008*a* ▶) *T*_min_ = 0.440, *T*_max_ = 0.746
5076 measured reflections
1966 independent reflections
1856 reflections with *I* > 2σ(*I*)
*R*_int_ = 0.032

#### Refinement

*R*[*F* ^2^ > 2σ(*F* ^2^)] = 0.021
*wR*(*F* ^2^) = 0.047
*S* = 1.02
1966 reflections
111 parameters
1 restraint
H atoms treated by a mixture of independent and constrained refinement
Δρ_max_ = 0.36 e Å^−3^
Δρ_min_ = −0.41 e Å^−3^
Absolute structure: Flack (1983 ▶), 690 Friedel pairs
Flack parameter: 0.048 (14)

### 

Data collection: *COLLECT* (Bruker, 2008 ▶); cell refinement: *DENZO-SMN* (Otwinowski & Minor, 1997 ▶); data reduction: *DENZO-SMN*; program(s) used to solve structure: *SHELXS97* (Sheldrick, 2008*b* ▶); program(s) used to refine structure: *SHELXL97* (Sheldrick, 2008*b* ▶); molecular graphics: *Mercury* (Macrae *et al.,* 2008 ▶); software used to prepare material for publication: *SHELXL97*.

## Supplementary Material

Crystal structure: contains datablock(s) I, global. DOI: 10.1107/S1600536812044765/fj2604sup1.cif

Structure factors: contains datablock(s) I. DOI: 10.1107/S1600536812044765/fj2604Isup2.hkl

Additional supplementary materials:  crystallographic information; 3D view; checkCIF report

## Figures and Tables

**Table 1:** Hydrogen-bond geometry (Å, °)

*D*—H⋯*A*	*D*—H	H⋯*A*	*D*⋯*A*	*D*—H⋯*A*
O1—H1⋯Br3^i^	0.75 (2)	2.49 (2)	3.232 (3)	175 (5)

Symmetry code: (i) 
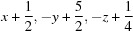
.

**Fig. 1.**
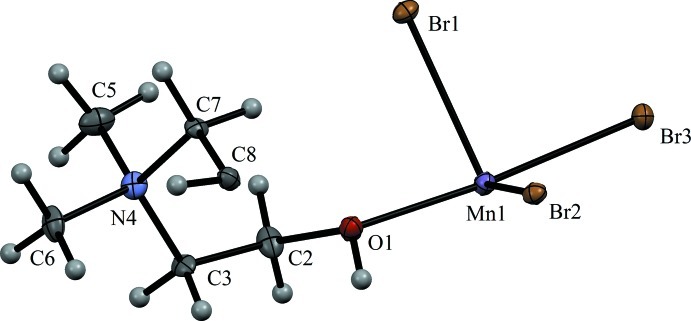
Asymmetric unit and labeling scheme of the title compound. Ellipsoids are presented at the 50% probability level.

**Fig. 2.**
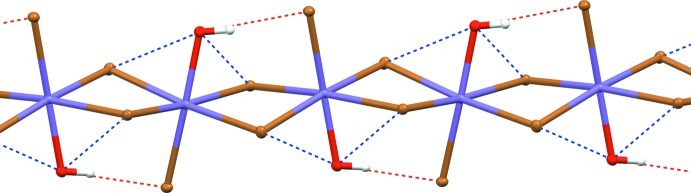
The one-dimensional linear chain with (µ-Br)2 bridges, Mn···Mn contact with a distance of 4.022 (1) Å and hydrogen bonding scheme.

**Fig. 3.**
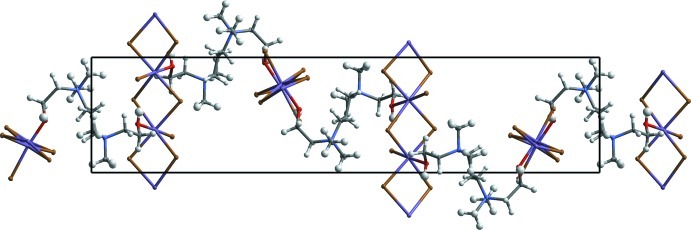
Undulated network formed by the L ligands connecting the alternating crossed Mn-(µ-Br)2—Mn chains, viewed along b-axis.

**Fig. 4.**
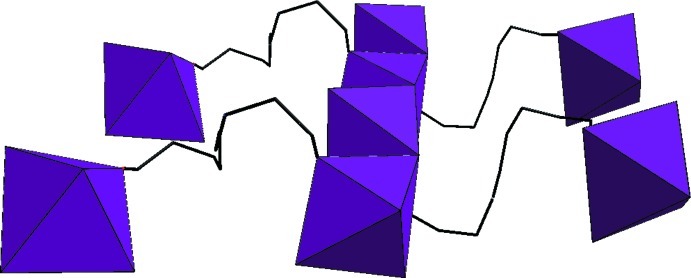
S-shaped conformation of the ligands (only ligand backbone showed) between the anti-parallel Mn-(µ-Br)2—Mn slightly distorted octahedron chains.

**Fig. 5.**
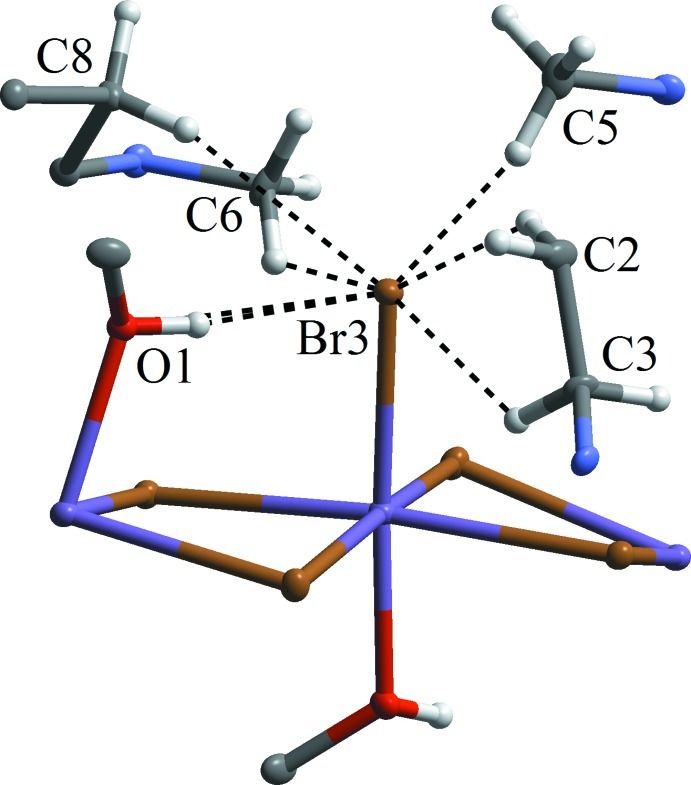
The structure is stabilized by weak intermolecular interactions between Br3 and nearby ligands.

**Fig. 6.**
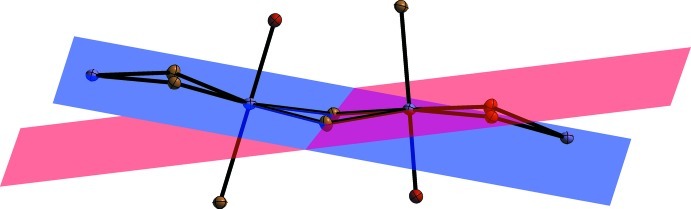
Zigzag tilting of the adjacent MnBr5O octahedra.

## supplementary crystallographic information

### Comment

Solid state chemistry of metal halides has been widely studied in order to improve various magnetic and non-linear optical applications. In the crystal structure of the type *MX*_4_*L*_2_, the bridging qualities of the halide anions and the coordination properties of the organic ligands result in various polymeric structures. For example, one-dimensional *M*-(µ-*X*)_2_—*M* bridged chains with low-dimensional arrangement have more suitable magnetic properties than classic structures of layered metal halide salts (Han *et al.* 2012; Wang *et al.* 2011 and Hitchcock *et al.* 2003).

The title compound, [Mn^II^(µ-Br)_2_µ-(C_11_H_28_N_2_O_2_)Br]_n_, crystallizes in a tetragonal *P*4_3_2_1_2 crystal system showing one Mn^II^ cation, half of a dicationic [C_11_H_28_N_2_O_2_]^2+^ ligand (*L*) and three bromide anions in an asymmetric unit (Fig. 1). In this three-dimensional polymer each Mn^II^ cation is coordinated by four bridging bromo anions in the equatorial plane. A single terminal bromo anion and a ligand are located in the axial positions of the distorted octahedron showing axial Br3—Mn1—O1 angle of 174.03 (8)°. The three-dimensional network structure comprises two alternating crossed Mn-(µ-Br)_2_—Mn chains (*a*- and *b*-axes) and an undulated L—Mn-(µ-Br)_2_—Mn—*L* chain (*c*-axis). Distances between parallel Mn-(µ-Br)_2_—Mn chains (planes through Mn -centres) are about 17.656 Å and between anti-parallel chains 8.7825 Å. This allows a formation of a structure model having alternating organic cation and a metal halide layers along *c*-axis (Figures 2 & 3).

In Mn^II^ cation coordination environment, the terminal Br^-^ anion fulfills the coordination of the Mn^II^ cation to octahedral MnBr_5_O. The metal–metal distance along the resulting chain of octahedra is 4.022 (1) Å. All the equatorial Mn—Br bridge bond distances are almost identical but still somewhat longer than the axial Mn1—Br3 bond. The bridging bromides and the adjacent Mn -centers form folded square-planar geometry, showing nearly orthogonal contact angle of 94.05 (2)° *via* Mn4—Br2- Mn4 atoms, and torsion angle of 12.82 (2)° through Mn4—Br2—Mn4—Br1 atoms.

In the structure, the ligands are in S-shaped conformation between the anti-parallel Mn-(µ-Br)_2_—Mn chains (Fig. 4). It seems that S-conformation is an ideal conformation for this type of relatively flexible ditopic ligand (Kärnä *et al.* 2010). The torsion angle C2—N4—N4—C2 is 156.70°. Similar cation conformations are found in ion pair structures [Zn^II^Br_4_(C_11_H_28_N_2_O_2_)] and (C_11_H_28_N_2_O_2_) Br_2_ H_2_O.

Classical Br3···H–O1 hydrogen bonds are present in the Mn^II^ cation coordination environment between the terminal Br^-^ anions of Mn^II^ cation and the hydroxyl group of the neighboring metal center (Fig. 5). Hence, it seems likely that in the parent complex the hydrogen bonding steers the oxygen's coordination to the Mn^II^ cation. Weak interactions between O1 and halide bridge on the other side of Br1 and Br2 leads to distortions of chains torsion angle. The angle between the Mn1—Br1—Br2 and Mn1—Br1—Br2 planes is 162.6°. For this reason, Mn-(µ-Br)_2_—Mn chains zigzag-conformation (Fig. 6).

### Experimental

The single crystals of the title compound were obtained in the following two steps: First, dicationic bromide salt, as the precursor, was synthesized in 30 ml of acetone by reacting 2.20 ml (13.15 mmol) of TMPDA, C_7_H_18_N_2_, and 2.16 ml (28.93 mmol) of 2-bromo-1-ethanol, C_2_H_5_BrO, for 48 h at 60 °C in a sealed flask. After removing the solvent, the white precipitation was washed by acetone and dried in *vacuo* (yield 71.6%; 3.58 g).

^1^H-NMR (DMSO, 250 MHz, p.p.m.): 2.08–2.32 (2*H*, m, CH_2_—CH_2_-CH_2_), 3.16 (12*H*, s, N—CH_3_), 3.31 (2*H*, s, H_2_O), 3.37–3.43 (4*H*, t, HO—CH_2_—CH_2_-N), 3.48–3.52 (4*H*, t, N—CH_2_-CH_2_—CH_2_-N), 3.84 (4*H*, s, CH_2_-OH), 5.29–5.33 (2*H*, t, OH)

Second, the precursor salt and the dried MnBr_2_ 4H_2_O (molar ratio ~1:1.5) were dissolved separately in minimum volume of warm methanol before combining the solutions. The title compound was synthesized in an open flask by metathesis reaction of the two aforementioned salts. The combined solution was stirred for about 1 h at 40 °C after which it was slowly cooled to RT and methanol was allowed to evaporate slowly. After several days, purple crystals suitable for X-ray analysis were formed.

### Refinement

Hydrogen atoms (except of a hydroxyl hydrogen atom that was taken from the electron density map) were calculated to their positions as riding atoms (C host) using isotropic displacement parameters that were fixed to be 1.2 or 1.5 times larger than those of the attached non-hydrogen atom.

### Crystal data

**Table:**

[Mn_2_Br_6_(C_11_H_28_N_2_O_2_)]	*D*_x_ = 2.370 Mg m^−^^3^
*M**_r_* = 809.69	Mo *K*α radiation, λ = 0.71073 Å
Tetragonal, *P*4_3_2_1_2	Cell parameters from 1871 reflections
Hall symbol: P 4nw 2abw	θ = 0.4–27.9°
*a* = 8.0163 (4) Å	µ = 11.69 mm^−^^1^
*c* = 35.3103 (18) Å	*T* = 123 K
*V* = 2269.1 (2) Å^3^	Block, violet
*Z* = 4	0.25 × 0.25 × 0.20 mm
*F*(000) = 1536	

### Data collection

**Table:**

Bruker–NoniusKappa APEXII diffractometer	1966 independent reflections
Radiation source: fine-focus sealed tube	1856 reflections with *I* > 2σ(*I*)
Graphite monochromator	*R*_int_ = 0.032
Detector resolution: 9 pixels mm^-1^	θ_max_ = 25.0°, θ_min_ = 2.8°
φ and ω scans	*h* = −9→9
Absorption correction: multi-scan (*SADABS*; Sheldrick, 2008*a*)	*k* = −4→9
*T*_min_ = 0.440, *T*_max_ = 0.746	*l* = −22→41
5076 measured reflections	

### Refinement

**Table:**

Refinement on *F*^2^	Secondary atom site location: difference Fourier map
Least-squares matrix: full	Hydrogen site location: inferred from neighbouring sites
*R*[*F*^2^ > 2σ(*F*^2^)] = 0.021	H atoms treated by a mixture of independent and constrained refinement
*wR*(*F*^2^) = 0.047	*w* = 1/[σ^2^(*F*_o_^2^) + (0.*P*)^2^] where *P* = (*F*_o_^2^ + 2*F*_c_^2^)/3
*S* = 1.02	(Δ/σ)_max_ = 0.001
1966 reflections	Δρ_max_ = 0.36 e Å^−^^3^
111 parameters	Δρ_min_ = −0.41 e Å^−^^3^
1 restraint	Absolute structure: Flack (1983), 690 Friedel pairs
Primary atom site location: structure-invariant direct methods	Flack parameter: 0.048 (14)

### Special details

**Table:**

Geometry. All s.u.'s (except the s.u. in the dihedral angle between two l.s. planes) are estimated using the full covariance matrix. The cell s.u.'s are taken into account individually in the estimation of s.u.'s in distances, angles and torsion angles; correlations between s.u.'s in cell parameters are only used when they are defined by crystal symmetry. An approximate (isotropic) treatment of cell s.u.'s is used for estimating s.u.'s involving l.s. planes.
Refinement. Refinement of *F*^2^ against ALL reflections. The weighted *R*-factor *wR* and goodness of fit *S* are based on *F*^2^, conventional *R*-factors *R* are based on *F*, with *F* set to zero for negative *F*^2^. The threshold expression of *F*^2^ > 2σ(*F*^2^) is used only for calculating *R*-factors(gt) *etc*. and is not relevant to the choice of reflections for refinement. *R*-factors based on *F*^2^ are statistically about twice as large as those based on *F*, and *R*- factors based on ALL data will be even larger.

### Fractional atomic coordinates and isotropic or equivalent isotropic displacement parameters (Å^2^)

**Table:**

	*x*	*y*	*z*	*U*_iso_*/*U*_eq_	Occ. (<1)
C2	0.8227 (5)	0.8960 (5)	0.15487 (11)	0.0158 (9)	
H2A	0.8379	0.8507	0.1290	0.019*	
H2B	0.7031	0.9236	0.1580	0.019*	
C3	0.8705 (5)	0.7633 (5)	0.18350 (10)	0.0143 (9)	
H3A	0.8340	0.6538	0.1735	0.017*	
H3B	0.9937	0.7605	0.1853	0.017*	
C5	0.6146 (5)	0.7687 (6)	0.22206 (11)	0.0197 (10)	
H5A	0.5825	0.6644	0.2095	0.030*	
H5B	0.5680	0.8634	0.2081	0.030*	
H5C	0.5713	0.7693	0.2480	0.030*	
C6	0.8652 (5)	0.6412 (5)	0.24628 (11)	0.0172 (10)	
H6A	0.8250	0.6531	0.2724	0.026*	
H6B	0.9874	0.6423	0.2461	0.026*	
H6C	0.8250	0.5356	0.2357	0.026*	
C7	0.8412 (5)	0.9495 (5)	0.24099 (11)	0.0116 (9)	
H7A	0.7764	0.9603	0.2647	0.014*	
H7B	0.8049	1.0396	0.2237	0.014*	
C8	1.0268 (5)	0.9732 (5)	0.2500	0.0140 (13)	
H8A	1.0594	0.9039	0.2720	0.017*	0.50
H8B	1.0961	0.9406	0.2280	0.017*	0.50
N4	0.8013 (4)	0.7825 (4)	0.22294 (9)	0.0130 (8)	
O1	0.9205 (3)	1.0470 (4)	0.15874 (8)	0.0140 (6)	
Br1	0.61915 (5)	1.32320 (5)	0.175357 (11)	0.01348 (10)	
Br2	1.13256 (5)	1.39037 (5)	0.167113 (10)	0.01205 (10)	
Br3	0.81111 (5)	1.55274 (5)	0.090982 (11)	0.01279 (11)	
Mn1	0.87136 (8)	1.27065 (7)	0.124710 (17)	0.01152 (14)	
H1	1.010 (3)	1.021 (5)	0.1575 (13)	0.017*	

### Atomic displacement parameters (Å^2^)

**Table:**

	*U*^11^	*U*^22^	*U*^33^	*U*^12^	*U*^13^	*U*^23^
C2	0.024 (2)	0.0120 (19)	0.011 (2)	−0.004 (2)	−0.003 (2)	−0.0030 (18)
C3	0.021 (2)	0.014 (2)	0.009 (2)	0.001 (2)	0.0015 (19)	0.0001 (17)
C5	0.013 (2)	0.025 (2)	0.021 (2)	−0.001 (2)	0.001 (2)	−0.006 (2)
C6	0.023 (2)	0.013 (2)	0.015 (2)	−0.0018 (19)	−0.0049 (19)	0.0016 (18)
C7	0.015 (2)	0.0074 (18)	0.012 (2)	−0.0020 (19)	−0.0027 (18)	−0.0025 (17)
C8	0.0114 (19)	0.0114 (19)	0.019 (3)	0.002 (3)	0.0015 (19)	0.0015 (19)
N4	0.0139 (16)	0.0157 (17)	0.0094 (17)	0.0011 (16)	−0.0017 (14)	0.0004 (15)
O1	0.0105 (14)	0.0137 (14)	0.0178 (15)	0.0010 (13)	0.0016 (14)	0.0026 (14)
Br1	0.01241 (19)	0.0168 (2)	0.01122 (19)	−0.00105 (19)	0.00124 (18)	−0.00200 (18)
Br2	0.01210 (19)	0.01371 (19)	0.01035 (19)	0.00068 (18)	0.00073 (16)	0.00004 (17)
Br3	0.01400 (19)	0.01200 (19)	0.0124 (2)	−0.00096 (19)	−0.00056 (18)	0.00178 (17)
Mn1	0.0117 (3)	0.0115 (3)	0.0113 (3)	−0.0001 (3)	0.0003 (3)	0.0014 (3)

### Geometric parameters (Å, º)

**Table:**

C2—O1	1.449 (5)	C7—C8	1.533 (5)
C2—C3	1.517 (5)	C7—H7A	0.9900
C2—H2A	0.9900	C7—H7B	0.9900
C2—H2B	0.9900	C8—C7^i^	1.533 (5)
C3—N4	1.507 (4)	C8—H8A	0.9900
C3—H3A	0.9900	C8—H8B	0.9900
C3—H3B	0.9900	O1—Mn1	2.194 (3)
C5—N4	1.501 (5)	O1—H1	0.748 (19)
C5—H5A	0.9800	Br1—Mn1	2.7319 (7)
C5—H5B	0.9800	Br1—Mn1^ii^	2.7635 (7)
C5—H5C	0.9800	Br2—Mn1^iii^	2.7407 (7)
C6—N4	1.491 (5)	Br2—Mn1	2.7472 (8)
C6—H6A	0.9800	Br3—Mn1	2.6010 (7)
C6—H6B	0.9800	Mn1—Br2^ii^	2.7407 (7)
C6—H6C	0.9800	Mn1—Br1^iii^	2.7635 (7)
C7—N4	1.517 (5)		
			
O1—C2—C3	112.7 (3)	C7^i^—C8—H8A	110.4
O1—C2—H2A	109.0	C7—C8—H8A	110.4
C3—C2—H2A	109.0	C7^i^—C8—H8B	110.4
O1—C2—H2B	109.0	C7—C8—H8B	110.4
C3—C2—H2B	109.0	H8A—C8—H8B	108.6
H2A—C2—H2B	107.8	C6—N4—C5	107.3 (3)
N4—C3—C2	116.8 (3)	C6—N4—C3	107.9 (3)
N4—C3—H3A	108.1	C5—N4—C3	109.9 (3)
C2—C3—H3A	108.1	C6—N4—C7	111.4 (3)
N4—C3—H3B	108.1	C5—N4—C7	106.5 (3)
C2—C3—H3B	108.1	C3—N4—C7	113.6 (3)
H3A—C3—H3B	107.3	C2—O1—Mn1	122.3 (2)
N4—C5—H5A	109.5	C2—O1—H1	106 (4)
N4—C5—H5B	109.5	Mn1—O1—H1	112 (4)
H5A—C5—H5B	109.5	Mn1—Br1—Mn1^ii^	94.082 (12)
N4—C5—H5C	109.5	Mn1^iii^—Br2—Mn1	94.254 (12)
H5A—C5—H5C	109.5	O1—Mn1—Br3	174.03 (8)
H5B—C5—H5C	109.5	O1—Mn1—Br1	84.28 (8)
N4—C6—H6A	109.5	Br3—Mn1—Br1	91.62 (2)
N4—C6—H6B	109.5	O1—Mn1—Br2^ii^	92.05 (8)
H6A—C6—H6B	109.5	Br3—Mn1—Br2^ii^	91.89 (2)
N4—C6—H6C	109.5	Br1—Mn1—Br2^ii^	84.75 (2)
H6A—C6—H6C	109.5	O1—Mn1—Br2	81.38 (8)
H6B—C6—H6C	109.5	Br3—Mn1—Br2	95.01 (2)
N4—C7—C8	113.7 (3)	Br1—Mn1—Br2	98.83 (2)
N4—C7—H7A	108.8	Br2^ii^—Mn1—Br2	172.12 (3)
C8—C7—H7A	108.8	O1—Mn1—Br1^iii^	89.94 (8)
N4—C7—H7B	108.8	Br3—Mn1—Br1^iii^	94.43 (2)
C8—C7—H7B	108.8	Br1—Mn1—Br1^iii^	173.07 (2)
H7A—C7—H7B	107.7	Br2^ii^—Mn1—Br1^iii^	91.67 (2)
C7^i^—C8—C7	106.5 (4)	Br2—Mn1—Br1^iii^	84.03 (2)
			
O1—C2—C3—N4	79.7 (4)	C2—O1—Mn1—Br2	179.1 (3)
N4—C7—C8—C7^i^	167.0 (4)	C2—O1—Mn1—Br1^iii^	−96.9 (3)
C2—C3—N4—C6	−179.3 (3)	Mn1^ii^—Br1—Mn1—O1	−105.44 (8)
C2—C3—N4—C5	64.0 (5)	Mn1^ii^—Br1—Mn1—Br3	78.92 (2)
C2—C3—N4—C7	−55.2 (5)	Mn1^ii^—Br1—Mn1—Br2^ii^	−12.838 (12)
C8—C7—N4—C6	54.1 (4)	Mn1^ii^—Br1—Mn1—Br2	174.24 (3)
C8—C7—N4—C5	170.8 (3)	Mn1^iii^—Br2—Mn1—O1	78.06 (8)
C8—C7—N4—C3	−68.1 (4)	Mn1^iii^—Br2—Mn1—Br3	−106.73 (2)
C3—C2—O1—Mn1	−175.3 (2)	Mn1^iii^—Br2—Mn1—Br1	160.85 (3)
C2—O1—Mn1—Br1	79.3 (3)	Mn1^iii^—Br2—Mn1—Br1^iii^	−12.785 (11)
C2—O1—Mn1—Br2^ii^	−5.2 (3)		

Symmetry codes: (i) −*y*+2, −*x*+2, −*z*+1/2; (ii) *x*−1/2, −*y*+5/2, −*z*+1/4; (iii) *x*+1/2, −*y*+5/2, −*z*+1/4.

### Hydrogen-bond geometry (Å, º)

**Table:**

*D*—H···*A*	*D*—H	H···*A*	*D*···*A*	*D*—H···*A*
O1—H1···Br3^iii^	0.75 (2)	2.49 (2)	3.232 (3)	175 (5)

Symmetry code: (iii) *x*+1/2, −*y*+5/2, −*z*+1/4.

## References

[bb1] Bruker (2008). *COLLECT* Bruker AXS Inc., Madison, Wisconsin, USA.

[bb2] Englert, U. & Schiffers, S. (2006). *Acta Cryst.* E**62**, m295–m296.

[bb3] Flack, H. D. (1983). *Acta Cryst.* A**39**, 876–881.

[bb4] Han, S., Liu, X.-Y., Cai, Z.-F. Z.-P., Yin, W.-T., Xie, X.-D., Zhou, J.-R., Yang, L.-M. & Ni, C.-L. (2012). *Inorg. Chem. Commun.* **24**, 91–94.

[bb5] Hitchcock, P. B., Lee, T. H. & Leigh, G. J. (2003). Dalton Trans. pp. 2276–2279.

[bb6] Hu, C. & Englert, U. (2006). *Angew. Chem. Int. Ed. Engl.* **45**, 3457–3459. 10.1002/anie.200504460 16622890

[bb7] Kärnä, M., Lahtinen, M., Hakkarainen, P.-L. & Valkonen, J. (2010). *Aust. J. Chem.* **63**, 1122–1137.

[bb8] Macrae, C. F., Bruno, I. J., Chisholm, J. A., Edgington, P. R., McCabe, P., Pidcock, E., Rodriguez-Monge, L., Taylor, R., van de Streek, J. & Wood, P. A. (2008). *J. Appl. Cryst.* **41**, 466–470.

[bb9] Otwinowski, Z. & Minor, W. (1997). *Methods in Enzymology*, Vol. 276, *Macromolecular Crystallography*, Part A, edited by C. W. Carter Jr & R. M. Sweet, pp. 307–326. New York: Academic Press.

[bb10] Sheldrick, G. M. (2008*a*). *SADABS* University of Göttingen, Germany.

[bb11] Sheldrick, G. M. (2008*b*). *Acta Cryst.* A**64**, 112–122. 10.1107/S0108767307043930 18156677

[bb12] Turgunov, K. K., Wang, Y., Englert, U. & Shakhidoyatov, K. M. (2011). *Acta Cryst.* E**67**, m953–m954. 10.1107/S1600536811022471 PMC3151818 21836934

[bb13] Wang, Y.-Q., Sun, Q., Yue, Q., Cheng, A.-L., Song, Y. & Gao, E.-Q. (2011). *Dalton Trans.* **40**, 10966–10974. 10.1039/c1dt10977d 21915428

